# Outcome-based reimbursement in Central-Eastern Europe and Middle-East

**DOI:** 10.3389/fmed.2022.940886

**Published:** 2022-09-23

**Authors:** Ildikó Ádám, Marcelien Callenbach, Bertalan Németh, Rick A. Vreman, Cecilia Tollin, Johan Pontén, Dalia Dawoud, Jamie Elvidge, Nick Crabb, Sahar Barjesteh van Waalwijk van Doorn-Khosrovani, Anke Pisters-van Roy, Áron Vincziczki, Emad Almomani, Maja Vajagic, Z. Gulsen Oner, Mirna Matni, Jurij Fürst, Rabia Kahveci, Wim G. Goettsch, Zoltán Kaló

**Affiliations:** ^1^Center for Health Technology Assessment, Semmelweis University, Budapest, Hungary; ^2^Division of Pharmacoepidemiology and Clinical Pharmacology, Utrecht Institute for Pharmaceutical Sciences, Utrecht University, Utrecht, Netherlands; ^3^Syreon Research Institute, Budapest, Hungary; ^4^National Health Care Institute, Zorginstituut Nederland, Diemen, Netherlands; ^5^The Dental and Pharmaceutical Benefits Agency, Tandvårds- och Låkemedelsförmånsverket, Stockholm, Sweden; ^6^National Institute for Health and Care Excellence, London, United Kingdom; ^7^Faculty of Pharmacy, Cairo University, Cairo, Egypt; ^8^Department of Medical Advisory and Innovation, Centraal Ziekenfonds (CZ) Health Insurance, Tilburg, Netherlands; ^9^National Health Insurance Fund of Hungary, Nemzeti Egészségbiztosítási Alapkezelõ, Budapest, Hungary; ^10^Department for Health Technology Assessment, Jordanian Royal Medical Services, Amman, Jordan; ^11^Croatian Health Insurance Fund, Zagreb, Croatia; ^12^Social Security Institution of Turkey, Ankara, Turkey; ^13^Social Security Main Office, Caisse Nationale de la Sécurité Sociale, Beirut, Lebanon; ^14^Department of Drugs, Health Insurance Institute of Slovenia, Ljubljana, Slovenia; ^15^Pharmaceutical Policies and Governance, Management Sciences for Health, Kyiv, Ukraine

**Keywords:** managed entry agreement, reimbursement, pricing, value-based pricing, health technology assessment, pay-for-performance, outcome-based agreement, outcome-based reimbursement

## Abstract

Outcome-based reimbursement models can effectively reduce the financial risk to health care payers in cases when there is important uncertainty or heterogeneity regarding the clinical value of health technologies. Still, health care payers in lower income countries rely mainly on financial based agreements to manage uncertainties associated with new therapies. We performed a survey, an exploratory literature review and an iterative brainstorming in parallel about potential barriers and solutions to outcome-based agreements in Central and Eastern Europe (CEE) and in the Middle East (ME). A draft list of recommendations deriving from these steps was validated in a follow-up workshop with payer experts from these regions. 20 different barriers were identified in five groups, including transaction costs and administrative burden, measurement issues, information technology and data infrastructure, governance, and perverse policy outcomes. Though implementing outcome-based reimbursement models is challenging, especially in lower income countries, those challenges can be mitigated by conducting pilot agreements and preparing for predictable barriers. Our guidance paper provides an initial step in this process. The generalizability of our recommendations can be improved by monitoring experiences from pilot reimbursement models in CEE and ME countries and continuing the multistakeholder dialogue at national levels.

## Introduction

Lower income countries (LICs) generally have a worse health status than the more affluent countries according to various metrics (1–5). This goes hand in hand with financing issues and as a result a limited access to the more expensive innovative health technologies (6–9). When there is a perceived unmet medical need, patient groups and the general public may strongly advocate for access despite the immense financial burden on the healthcare budgets in various LICs: from the Central and Eastern European (CEE) region to the LICs of the Middle East (ME), and several other parts of the world as well.

One possible solution to bridge this gap are the various types of confidential agreements between payers and manufacturers, known as managed entry agreements (MEAs) (10–12). MEAs can be considered well-balanced compromises between the aforementioned two stakeholder groups (13) and have shown promising results in granting access to innovative pharmaceuticals in Western European (WE) countries for example (14–16).

Outcome-based agreements are a subtype of MEAs (17, 18), that link payments through various ways to the health benefits that patients realize due to the use of the novel health technology (including pay-for outcome, conditional treatment continuation, and coverage with evidence development). These can effectively reduce the risk of payers in cases when there is important uncertainty or heterogeneity regarding the clinical value of the pharmaceutical in question (19). Outcome-based MEAs play an important role in the healthcare financing of several WE countries, for example in Italy (20). However, their uptake in LICs seems to be lagging behind (21) as these countries often rely more on simpler methods, such as financial managed entry agreements and volume restrictions (such as price-volume agreement, manufacturer funded initial treatment period, utilization cap, etc.), without accomplishing the potentially increased patient access due to outcome-based agreements (19).

HTx is a Horizon 2020 project supported by the European Union for from 2019 to 2024. The main aim of HTx is to create a framework for the Next Generation Health Technology Assessment (HTA) to support patient-centered, societally oriented, real-time decision-making on access to and reimbursement for health technologies throughout Europe. Task 4.4 of the HTx project is dedicated to payment models and sustainable healthcare funding. A key result of this task has been the publication of a feasibility analysis of the application of MEAs for innovative therapies (22).

As part of the European Commission funded HTx H2020 project, the objective of this study was to explore the transferability of outcome-based payment methods within and outside the European Union with a special focus on countries in the CEE region and LICs from the ME. This research aims to highlight the most important barriers that prevent the widespread use of these agreements, and to recommend potential solutions to the identified barriers.

## Methods

**FIGURE 1:**
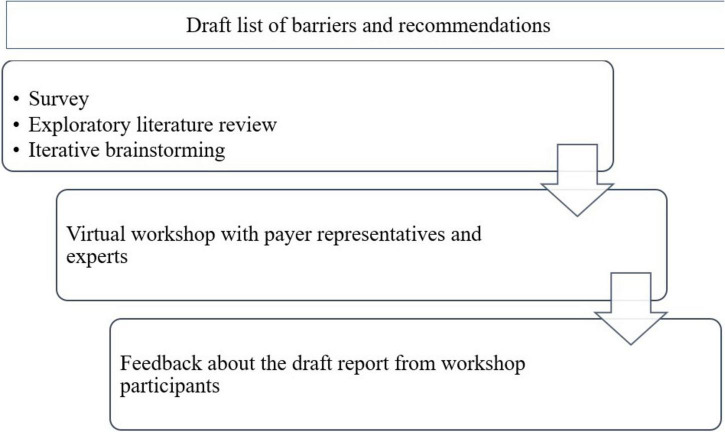
Process of creating policy recommendations for the implementation of outcome-based reimbursement models for technologies with high upfront cost in Central and Eastern European and Middle Eastern countries.

Our research project is the continuation of an overview on different types of MEAs and payment mechanisms for innovative therapies (22). The first step in our research was the collection of information from relevant literature and the HTx network regarding the potential barriers and solutions for implementing outcome-based reimbursement models in CEE and in the ME (see Figure 1). Information collection was carried out in parallel through three different sources. Information about utilization status and potential barriers of outcome-based agreements in these countries was collected through a survey, which covered four topics on outcome-based reimbursement and payment models, including one topic on use of reimbursement models such as discounts, pay for outcome, etc. The results of the survey were described in a different manuscript (23). A targeted review of scientific and gray literature was carried out in parallel with the survey, to identify and explore further barriers and potential solutions for the implementation of outcome-based reimbursement models. During iterative rounds of discussions with HTx consortium members representing different stakeholders in the HTA arena, the list of barriers and recommendations were expanded with their insights. The information from these sources was reviewed continuously with the objective minimizing overlaps in the list of barriers and streamlining recommendations relating to barriers. Considering the parallel and iterative nature of the exploratory process for these barriers and related recommendations, the clear back-tracking to identify the various sources would be cumbersome as well as irrelevant for the next steps.

The second step in our approach was to review the draft list of barriers and recommendations identified in the earlier step, with representatives of health care payers and with health care financing experts (advisers of health care payers or former payers) from CEE and ME countries during a policy workshop. Considering the travel restrictions related to the COVID-19 pandemic, the workshop was organized as a virtual meeting. The virtual workshop took place in June 2021 with 16 members of the HTx consortium and 14 payer experts, from Bulgaria, Croatia, Hungary, Poland, Ukraine, Serbia, Slovakia, Slovenia, and Turkey (9 CEE countries); Egypt, Jordan, and Lebanon (3 ME countries); and the Netherlands, Sweden and the United Kingdom (3 WE countries).

In the first part of the workshop, participants received an introduction of relevant experiences in Sweden, United Kingdom, and Netherlands. This was followed by a presentation of the draft list of barriers and recommendations created by the research team. Participants were then allocated into working groups, consisting of four payer experts and four representatives from the research team in each group. Finally, the rapporteurs summarized the findings of each working group, providing feedback to workshop participants, identifying common themes, and clarifying all emerging questions.

As part of the third and final step of our approach, the research team summarized their findings in a draft report containing the consolidated list of barriers and recommendations identified. Workshop participants were given an opportunity to make final comments and amendment suggestions to the report. After the workshop, we reached out to participating experts to confirm their inputs provided during the workshop.

The final outcome of the research carried out by the HTx team was a list of barriers and potential solutions for outcome-based reimbursement models, based on a consensus among the research team and workshop participants. In the course of this project, list of barriers and recommendations for implementing delayed payment schemes were also described in a separate manuscript (24).

## Results

After deduplication of barriers retrieved from different sources, the HTx research team identified 20 different barriers in five broader groups: (i) transaction costs and administrative burden, (ii) measurement issues, (iii) information technology (IT) and data infrastructure, (iv) governance, and (v) perverse policy outcomes. Practical recommendations were drafted to address these barriers (see summary in Table 1).

**TABLE 1:** Summary of barriers and recommendations for health care payers for the implementation of outcome-based reimbursement models.

Group of barriers	Barriers	Recommendations
Transaction costs and administrative burden	Complex and resource intensive negotiations on contractual terms (including the first agreement and renegotiations)	Consider transferring the structure of existing agreements from higher income countries Develop contract archetypes for most common schemes Include re-opener clause into the agreements When agreements are renegotiated, the latter agreement should be simpler than the first
	Costly collection of outcomes data without appropriate funding mechanism for data collection	If feasible, rely on existing infrastructure reuse of existing medical or claims data cost of incremental data collection should be covered by pharmaceutical manufacturers
	Administrative burden on health care providers to collect data	Health care institutions should opt-in to prescribe medicines in outcome-based schemes Involve leading centers in a network to publication of real world data
Measurement issues	Lack of HE&OR expertise to specify and determine treatment effects in non-randomized and observational settings (especially in rare diseases)	Capacity building in HE&OR (including education and collaboration in international initiatives)
	Long-time frame to capture hard end-points, however, in surrogate outcomes may not guarantee improvement in hard endpoints	Greater dialogue between clinical opinion leaders, HE&OR experts, payers, and patient representatives capturing different perspectives both at the initiation and follow-up of agreements Surrogate endpoints should be valid predictors of patient outcomes. If such validation is not available upfront, additional data collection within the agreement can be considered to validate the surrogate outcome
	Treatment success is affected by confounding factors that cannot be controlled (e.g., inefficient health systems, local practice patterns, or poor treatment adherence)	Outcome-based agreements provide incentives to manufacturers to address inefficiencies of health care delivery
IT and data infrastructure	Failure to capture the necessary data to reduce uncertainty within current infrastructure	If difficulties to collect data is expected, consider a pilot phase with adjustment according to early experiences Terminate the agreement, if there is no better solution
	Fragmentation of healthcare financing and service provision makes it difficult to undertake outcome-based schemes	In fragmented health care system limit the scope of outcomes to hard end-points Promote national platform for outcome based agreements with system based incentives even in fragmented health care systems
	Limited compatibility of medical, pharmacy, and payer data systems restrict meaningful retrospective analysis	Invest into building pragmatic MEA implementation frameworks by linkage of databases reuse of existing data
	Limited uptake of patient registries	Facilitate the establishment of patient registries with incentives to all stakeholders
Governance	Lack of regulation	Consider the implementation of pilot cases Consider rationale selection mechanism when to apply outcome based agreements Prepare regulatory legal framework based on experiences in the pilot phase
	Incentives of health care professionals, patients, and manufacturers to improve patient access limits their compliance to keep agreements	Outcomes should be objective, clearly defined, reproducible, and difficult to manipulate
	Unknown consequences of better results than expected (e.g., can prices be increased?)	No special action is needed similarly to current practice outside outcome based agreements such situation rarely happen, as clinical benefits measured in clinical trials can hardly be replicated in real world
	Limited trust between payers and manufacturers	Outcomes data should be made available for independent audit Sales are frozen and be made available depending on the outcome to the payer or to the manufacturer
	Difficulties for health authorities to delist health technologies or renegotiate prices	Clear legal foundation to support delisting of medicines due to limited efficacy (similarly to existing safety issues) Involve clinical and patient representatives into delisting decisions
Perverse policy outcomes	Equity in patient access may be compromised when the new technology is available only in selected centers	Consider that no agreement would result in no patient access to new technologies Extend the scope of prescribing centers when renegotiating the agreement
	No improvement in the evidence based of health technologies, if real world data in outcome-based schemes remains unpublished	Evidence-gathering efforts can be shared and implemented jointly by countries to improve information quality and completeness and to counter potential information bias Evidence about the effectiveness of health technologies should be considered a global public good. Publication of real-world evidence in outcome based agreements should be an international standard
	Non-transparency of policy decisions due to confidential nature of data captured in agreements	Increase transparency around key components of the scheme
	Difficulties to implement value based health care, as due to confidentiality of actual prices, true cost-effectiveness of any health care interventions cannot be calculated	Public availability of HTA documents Two-way sensitivity analysis for the prices of compared technologies in economic evaluations
	Lower income countries may pay more for medicines, as higher income countries potentially have greater economic power when negotiating about confidential discounts	Strengthen HTA system to calculate the local value based price Consider joint procurement by lower income countries

### Recommendations for barriers of implementing outcome-based reimbursement models in Central and Eastern Europe and Middle East countries

#### Transaction costs and administrative burden

Unlike MEAs with financial terms only the transaction costs and administrative burden of implementing outcome-based agreements are more significant.

##### Complex and resource intensive negotiations on contractual terms

Outcome-based reimbursement models require complex and resource intensive negotiations on contractual terms. The complexity of negotiations can be reduced by considering transferring the structure of existing agreements from higher income countries. Multinational manufacturers, supranational organizations or international consortiums supported from the Horizon Europe framework program can facilitate the transfer of agreements by developing contract archetypes for the most common health care financing systems. Contracts should have clarity on foreseeable problems, for example a re-opener clause should be a standard inclusion to manage situations when a new product is entering the market. Finally, in parallel with the increasing evidence base of technologies, when agreements are renegotiated, the new agreement should be simpler than the original. There is limited benefit from maintaining unnecessarily complex agreements as the uncertainty is reduced over time.

##### Costly collection of outcomes data without appropriate funding mechanism for data collection

The implementation of an outcome-based reimbursement model may itself require significant financial resources. Therefore, it is important to resolve how the associated costs can be minimized and who should bear the responsibility of financing the additional data collection (25).

If feasible, payers should rely on the existing infrastructure that would minimize extra costs. For example, existing medical or reimbursement claims data could contribute to data collection without imposing extra costs. If these do not provide enough evidence, additional data collection is inevitable. The underlying costs should be covered by the pharmaceutical manufacturers. Payers should make sure that the pharmaceutical manufacturers take responsibility for the required extra data collection (and associated costs). It is also suggested that the data will be made publicly available.

##### Administrative burden on health care providers to collect data

Typically, an outcome-based agreement will require relevant real-world data to be collected to resolve uncertainties that remain from the trial evidence. This cannot be achieved without the involvement of health care providers. However, in usual clinical practice, data on treatment outcomes are not collected in the highly structured way that is typical of randomized clinical trials. Therefore, usually a significant commitment is required from the healthcare providers to collect data during the treatment (26).

Payers should incentivize healthcare institutions to prioritize the success of outcome-based agreements, such as financial or other rewards. Healthcare institutions should opt-in to prescribe medicines within the relevant outcome-based schemes. This means that they actively commit to collecting data and, in return, become eligible to prescribe the medicines only that way. Furthermore, involving leading centers in a network to collect and publish real-world data could also contribute to minimizing the administrative burden on health care providers.

#### Measurement issues

The internal validity of real-world evidence is less than the scientific evidence generated in randomized clinical trials, which are the gold standard way to measure relative treatment effectiveness. On the other hand, the external validity of real-world evidence is greater, because the data reflect actual clinical practice much more closely than clinical trials. Hence, with careful measurement of real-world outcomes in payment agreements valuable complementary data can be generated, which ultimately can address the uncertainties regarding new technologies.

##### Lack of health economics and outcomes research expertise

Specifying and determining treatment effects in non-randomized and observational settings are critical for outcome-based agreements. However, these processes are resource intensive and very specialized. Therefore, an important step is to enable the training of payers and their advisors about health economics and outcomes research (HE&OR). In addition, the capacity of HE&OR experts should be increased in payer organizations. Capacity building can be facilitated by participation in international educational initiatives. Finally, the capacity constraints in HE&OR can also be reduced by implementing joint outcome-based reimbursement models at the regional level.

##### Surrogate outcomes are not warranties

Since a long timeframe is often needed to capture hard endpoints like survival, surrogate outcomes are usually the best-available measures to support reimbursement decisions in the immediate term (27). However, surrogate outcomes may not guarantee improvements in more important hard endpoints (28).

In outcome-based reimbursement models, surrogate endpoints should only be considered suitable for data collection when they have been demonstrated to be valid predictors of long-term patient outcomes. If such validation is not available upfront, additional data collection within the outcome-based reimbursement model can be considered to validate the surrogate outcome. A greater dialogue between clinical opinion leaders, HE&OR experts, payers and patient representatives can ensure that appropriate outcomes are included in data collection for outcomes-based agreements. Furthermore, over time, such dialogues will increase the HE&OR knowledge and expertise in CEE and ME payer organizations.

##### Confounding factors of the treatment success

There are several factors that may impact the outcome, for instance inefficiencies in the health care system, and confounding factors, such as poor adherence of patients, suboptimal patient pathways or hidden access barriers to supplementary services (29). Outcome-based reimbursement models create direct incentives to manufacturers to optimize patient selection and to support health care providers in side-effect management and educating patients. Partnership between the payers and manufacturers in monitoring and improving health outcomes is recommended as that can contribute to reducing the inefficiency of health care delivery.

Given that there is a real human and financial resource restriction, outcome-based agreements should not be the standard when simpler models can suffice (e.g., price-volume agreement). A very clear selection mechanism should be developed and implemented to make sure outcome-based agreements are applied rationally and sparingly.

#### IT and data infrastructure

As the current way of treatment and the underlying financing mechanisms are not set for measuring and reporting real-world health outcomes, IT and data infrastructure can be a barrier for implementing outcome-based reimbursement models.

##### Failure to capture necessary data

As outcome-based payments are usually not based on health outcomes, the current IT infrastructure of health care payers is mainly designed to collect and monitor electronic utilization records of health services and technologies. Hence, failure to capture necessary outcomes data is a real concern when implementing outcome-based reimbursement models (30).

If such difficulties are expected, a pilot phase of implementing outcome-based reimbursement models can be rolled-out to assess the feasibility and adjust the agreements. However, if an adjustment still does not provide a better solution, terminating the outcome-based agreement can be considered.

##### Fragmented health care financing and service provision

Outcome-based reimbursement models can be more challenging in health care systems with multiple payers. An ongoing agreement should not prevent patients from choosing another health care payer; however, such a change may complicate the outcome-based agreement. In addition, in some countries patients may have duplicate coverage, and so they can choose which is the simpler option for getting reimbursement to specific healthcare services or technologies. For instance, if the medicine is covered by the public payer and the advanced monitoring and diagnostics through a private supplementary insurance, there might be a disconnect between the therapy and outcomes.

In health systems with fragmented healthcare financing, limiting the scope of outcomes to hard endpoints can facilitate the feasibility of implementing outcome-based reimbursement schemes. Besides, promoting the national platform (e.g., coordinating center for outcome-based agreements serving for multiple payers) with system-based incentives could contribute to a successful agreement scheme. Therefore, it is recommended that outcome-based agreements are centrally coordinated, even in a fragmented system, and implemented with system-based incentives.

##### Limits in compatibility of system data

Usually medical, pharmacy and payer data systems are designed for different purposes, and therefore often possess different data structures and codes. Limited compatibility of data from the different systems could reduce the potential of data collection for outcome-based schemes (31).

A general framework for the compatibility of health care data is key in implementing outcome-based data collection. Linkage of medical records, patient registries and payers’ databases in federated data networks using a common data model (see European Health Data Evidence Network, EHDEN) and reusing existing data can be an answer to the increasing need of real-world evidence for multiple research questions (32). The payers in LICs should invest in linking the different data sources or require pharmaceutical manufacturers to pay for additional data collection.

##### Limited uptake of patient registries

Patient registries are the key, especially for rare diseases with high treatment cost (33). Although this is clear to all stakeholders, due to the barriers–such as lack of physical and financial resources–countries are not setting up registries to all relevant patient groups. The potential benefit factors of such registries should be identified and encouraged to facilitate the establishment of comprehensive disease registries which are preferably aligned internationally.

#### Governance

Implementation of financial MEAs, which are common even in LICs (21, 34), would not be possible without addressing the regulatory and legal perspectives. An established regulatory and legal framework empowers both the payers as well as the manufacturers and speeds up contract negotiations.

##### Lack of regulation

The minimum criterion is to enable the possibility for healthcare payers to conclude outcome-based reimbursement models with the manufacturers in the legislative and regulatory framework (35).

Considering pilot outcome-based reimbursement schemes in the initial period would create an opportunity for more sustainable regulation. Based on the experience of the pilot cases a regulatory and legal framework should be proposed with recommendation for a rationale selection mechanism on when to apply outcome-based reimbursement models. Besides, international collaboration on how to regulate the implementation of outcome-based reimbursement models would be beneficial. LICs can learn from each other especially if health systems are similar. Regional collaboration is a good opportunity to overcome the barrier of lack of regulation.

##### Contradicting motivation of limiting patient access

Defining strict inclusion criteria and complying with the rules is crucial for the payers but is perhaps less important for the other stakeholders. Healthcare professionals, patient groups and manufacturers may advocate for unconditional patient access or might find it difficult to comply with the established agreements. For instance, healthcare providers may find it difficult to stop a treatment for their patient, even if the pre-specified outcome is not reached and the medicine failed to show meaningful clinical benefit. It is therefore important that the outcome is clearly defined, can be objectively and independently measured so that it is reproducible and difficult to manipulate. Furthermore, the rationale for terminating or restricting treatment should be clearly communicated; that is, following additional data collection, the new treatment potentially causes more harm to the health care system than good.

##### Unknown consequences of better results

Although clinical benefits measured in clinical trials cannot easily be replicated in the real world, in theory, the therapy can result in worse, the same or better outcomes in the real world than in the clinical trial. This leads to the question what happens if results are better than expected. For example, can the manufacturer increase the price? For such cases, no special policy action is recommended, which has to be stated explicitly in the agreement, as better results can happen without outcome-based reimbursement models as well.

##### Limited trust between payers and manufacturers

Outcomes data of patients cannot be accessible for manufacturers (due to legal restrictions), which implies that health care payers have direct control over individual patient records with serious financial implications on manufacturers. In order to build mutual trust between payers and manufacturers the outcome data should be available for an independent audit. As the audit is requested by the manufacturer, its cost should also be covered by them. When needed, sales revenues or paybacks could be frozen until the audit confirms the outcome data, and the ring-fenced budget can be released after the audit is completed (36). In some cases, manufacturers may directly receive the aggregated data from the coordinating centers, which can be compared with the claims data in payers’ databases.

##### Difficulties in excluding therapies from reimbursement and renegotiating prices

Exclusion of high-cost therapies from the reimbursement list due to lower-than-expected health benefits is politically sensitive and controversial, as even in such cases the technology might be the only alternative for several patients. Although adjustment of the price to the lower clinical value may be possible, pharmaceutical companies are reluctant to lower drug prices due to market externalities through the external price referencing system. In such case confidential price reductions should be part of the outcome-based agreement. Similarly, to the regulatory response related to safety concerns, clear legal foundation is necessary to support delisting therapies from public reimbursement, if real-world health benefits are proven to be worse than expected. The relevant medical society and patient representatives should be involved in these decisions (25).

#### Perverse policy outcomes

Even in case of the best legislative framework, complex contractual agreements can have negative implications beyond the improvement in the agreed health outcomes. Such implications should be carefully evaluated prior to introducing the outcome-based schemes in partnership with all stakeholders.

##### Equity in patient access

If new therapies would be available only in those centers participating in the outcome-based reimbursement models, equitable patient access may be compromised. On the other hand, patient access in at least a few selected centers is still better than no patient access to the new technology without the outcome-based reimbursement model. An equitable geographical coverage should be considered when selecting the prescribing centers, both upfront as well as during each renegotiation phase.

##### No improvement if real world data remains unpublished

Outcome-based reimbursement models provide an opportunity to generate real-world evidence regarding technologies with uncertain health benefits. However, if real-world data collected in such scheme remains unpublished (37), there is no improvement in the evidence-base for those stakeholders who are not directly involved in the analysis of primary data or do not get access to aggregated results. Furthermore, not publishing the data leads to an inefficient duplication of efforts among payers facing similar uncertainties in similarly resource-constrained health care systems.

As noted above in Section “Transaction costs and administrative burden” the implementation of an outcome-based reimbursement model can be expensive, especially if the real-world evidence generated in such agreements remain unpublished, and consequently every payer has to undertake this effort individually. Therefore, it is highly recommended to publish and transfer the real-world evidence from early technology adopter countries to LICs with usually delayed launch of new technologies. A separate research of the HTx project aims to list the barriers and provide recommendations transferring real-world evidence to LICs (38).

Evidence gathering methods should be shared and implemented jointly by multiple healthcare payers in different countries. This would improve the quality and completeness of the data and prevent potential information bias. It should be highlighted that evidence about the effectiveness of health technologies should be considered a global public good, and so publishing real-world evidence from outcome-based agreements should be an international standard (39).

##### Non-transparency of policy decisions

Certain elements of outcome-based agreements, especially the net price with the actual paybacks, are considered confidential, which reduces the transparency of the resource allocation decisions. The public confidence in policy decisions can be improved by increased transparency around the key components of the scheme, for example publication of the objectives, process and structure of agreements and the generated real-world data (18). The importance of real-world evidence is constantly increasing in the field of policy decisions (40), and extension of the evidence base of new technologies from such agreements would be highly beneficial for all different stakeholders of the society.

##### True cost-effectiveness of health care interventions cannot be calculated

Implementing value-based healthcare is a challenge due to the confidentiality of actual prices, meaning the true cost-effectiveness of health technologies cannot be calculated (13). However, this problem is already well-known from experiences of financial MEAs, so the inclusion of outcome-based agreements to reimbursement models in countries with existing confidential price agreements would neither resolve nor introduce the problem. Two-way sensitivity analysis for the prices of compared technologies can make economic evaluations relevant to health care payers, who may have precise knowledge on the net prices of both the comparator and the new technology in their setting. Eventually, the complexity of cost-effectiveness calculations may even be reduced by publishing transparent HTA documents, with special focus on the newly generated real-world evidence.

##### Lower income countries pay more for medicines

Higher income countries usually have more resourceful HTA bodies and greater economic power when negotiating about confidential discounts, and so lower income countries may pay even more for medicines. The limited HTA capacity of late technology adopter lower income countries can be alleviated by re-using the transferable elements of joint HTA reports and focusing only the calculation of the local value-based price (41). The market potential of lower income countries can be increased if they establish a joint procurement process, which can compensate manufacturers with a larger volume in case of successful agreements.

## Discussion

Population health status is correlated with the economic status of countries, and so the capacity to benefit from innovative technologies may even be greater in lower income countries. However, the health gap between poorer and more affluent countries cannot be reduced, if policymakers in the healthcare sector of lower income countries do not put more emphasis on selecting only those technologies for reimbursement, which can generate greater absolute health gain. Proposals for outcome-based contracting platforms, with the goal of aligning the interests of all stakeholders, have been considered recently (42). The opportunity cost of the selection process for high-cost technologies can be mitigated by implementing outcome-based reimbursement models, in which the health gain is guaranteed. In other words, healthcare payers should have the opportunity to purchase health instead of purchasing health technologies. Such agreements may contribute to new standards in health care provision, in which health gain has primary importance over other objectives for healthcare providers, patients, pharmaceutical and medical device manufacturers. The importance of reaching target health gain creates incentives for all stakeholders to pay more attention to health outcomes not only by creating access, prescribing and utilizing new health technologies, but also by streamlining patient pathways and improving other elements of care.

The momentum for outcome-based reimbursement models is strengthened by the ongoing initiative to enhance regulatory post-authorization requirements, especially in those cases where only conditional market authorization is granted for new medicines which respond to huge unmet medical needs with uncertain clinical value (43).

Implementation of outcome-based reimbursement models is challenging, especially in resource constrained health care systems of lower income countries. However, those challenges can be faced by health care systems committing to testing them in pilot agreements, and actively preparing for predictable barriers.

Implementing outcome-based reimbursement models can be beneficial to all stakeholders for different reasons. For health care payers it can result in reduced uncertainty of policy decisions. Patients and caregivers could benefit from earlier patient access due to outcome-based agreements, without disincentives to understand about the pros and cons of new health technologies. The clinicians could confirm the real-world benefits of health technologies to subgroups of patients, which ultimately increases the evidence base of therapeutic decisions. The manufacturers could also address appropriate response to payers’ concerns about premature scientific evidence, which ultimately has the potential to accelerate market access and justify premium price of high-value medicines.

Our guidance paper is an initial step in this process. The generalizability of our recommendations can be improved by monitoring experiences from pilot reimbursement models in CEE and ME countries and continuing the multistakeholder dialogue at country levels. While this guidance was developed with payer experts from many countries, continued dialogue should include representatives of settings and stakeholders that were not represented here partly due to the pandemic period.

## Data availability statement

The original contributions presented in this study are included in the article/supplementary material, further inquiries can be directed to the corresponding author.

## Author contributions

All authors listed have made a substantial, direct, and intellectual contribution to the work, and approved it for publication.
